# 

**Published:** 1886-10

**Authors:** 


					﻿a.	- .-*»«*. ^afcw . ^•A»	~.«Bk. .in*. . »!■
KMerrii at th- h.*t (tftir.'tlt J .. .<•>», 7’> <<>'.<’> ,S'« ’■•■n-t-t 'hint Malin-.
1 V°L- 11 )
/OCTOBEE, 188S.
W®**-
JL»"*^» jL	JLa
MEDICAL 
twftwe
F. E. DANIEL, M. 1)., Editor and Publisher, AUSTIN, TEXAS.
sokthi.hx office, 1«> filbert st.. Philadelphia.
Warner & Draughon, Steam Printers, Austin, Texas.
				

